# Electrodeposited ZnO/Zn(OH)_2_ Nanosheets as a Functional Interface for Dendrite‐Free Lithium Metal Anodes

**DOI:** 10.1002/smll.202503607

**Published:** 2025-05-30

**Authors:** Da‐Eun Hyun, Jong Chan Choi, Yoon Ho Kim, Yejin Ra, Jae Sol Sim, Jung‐Kul Lee, Yun Chan Kang

**Affiliations:** ^1^ Department of Materials Science and Engineering Korea university 145 Anam‐ro Seongbuk‐gu Seoul 02841 Republic of Korea; ^2^ Department of Chemical Engineering Konkuk university Hwayang‐dong Gwangjin‐gu Seoul 143–701 Republic of Korea

**Keywords:** current collector modification, electrodeposition method, lithium metal anode, solid electrolyte interphases, ZnO/Zn(OH)_2_ nanosheets

## Abstract

Modifying the current collector is a promising strategy to enable Li metal anodes with minimal Li consumption. Herein, a scalable electrodeposition method is introduced to construct 3D ZnO/Zn(OH)_2_ nanosheets on Cu foil (ZOH NSs–Cu foil). Cu(OH)_2_ nanowires are first formed via anodization, followed by electroconversion of Cu^2+^ and Zn^2+^ ions. DFT calculations reveal that the ZOH NSs–Cu foil exhibits high Li adsorption energy, imparting strong lithiophilicity and lowering the Li nucleation overpotential. The 3D nanosheet structure provides a large electrochemically active surface, reducing the effective current density. Furthermore, ZOH NSs–Cu foil exhibits low charge transfer resistance and promotes a Li_2_O/LiF‐rich solid electrolyte interphase (SEI) layer, further reducing interfacial resistance. SEM analysis and simulations confirm uniform Li deposition on ZOH NSs–Cu foil. In asymmetric cells (1 mAh cm^−2^ at 1 mA cm^−2^), ZOH NSs–Cu foil supports stable cycling for over 400 cycles. Furthermore, a full cell coupling a LiFePO_4_ (LFP) cathode with a Li@ZOH NSs–Cu foil anode retains high capacity with ≈100% Coulombic efficiency over 350 cycles at 1 C, even at an N/P ratio of ≈1.9. This binder‐free, scalable approach offers precise Li deposition control and excellent electrochemical performance, advancing the practical application of Li metal anodes.

## Graphical abstract

A scalable electrodeposition method is developed to fabricate 3D ZnO/Zn(OH)_2_ nanosheets (ZOH NSs) on Cu foil. The ZOH NSs exhibit high lithiophilicity, lowering the nucleation overpotential and enabling uniform Li deposition. This facilitates robust SEI layer formation, which lowers charge transfer resistance and enhances the cycling stability of Li metal anodes.

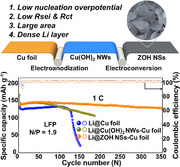

## 1 Introduction

The demand for advanced battery systems with capacities and energy densities higher than the theoretical limits of Li‐ion batteries (LIBs) is steadily increasing. Li metal has emerged as a standout next‐generation anode material owing to its ultrahigh theoretical specific capacity (3860 mAh g^−1^, ≈10 times greater than that of graphite), low redox potential (−3.04 V vs the standard hydrogen electrode), and exceptionally low mass density (0.53 g cm^−3^):^[^ ^1^, ^2^, ^3^, ^4^, ^5^ ^]^ however, the growth of Li dendrites and significant volume changes can occur during repetitive Li plating and stripping processes owing to the high reactivity of Li metal, inhomogeneous Li^+^ flux distribution, and its frameless nature.^[^ ^6^, ^7^, ^8^ ^]^ These issues shorten the cycle life of batteries and that significantly imped the commercialization of Li metal anodes (LMAs).

Several strategies have been developed to suppress Li dendrite growth and address interface challenges, including the use of 3D hosts,^[^ ^9^, ^10^, ^11^ ^]^ modified current collectors (CCs),^[^ ^12^, ^13^, ^14^ ^]^ advanced electrolyte additives,^[^ ^15^, ^16^, ^17^ ^]^ and artificial solid electrolyte interphase (SEI) layers.^[^ ^18^, ^19^, ^20^ ^]^ Although the thick Li metal layers or bulky 3D hosts used to modify LMAs can achieve excellent cycling performance, they often fail to balance capacity with the negative/positive electrode capacity (N/P) ratio, thereby wasting significant Li resources and reducing energy density.^[^ ^21^, ^22^ ^]^ Modifying the CCs enables the electrodeposition of Li metal, which affords precise control over the N/P ratio, enabling the deposition of Li metal layers of various thicknesses as required, including anode‐free configurations.

Cu foil is the most common anode CC used in LIBs owing to its lightweight, low cost, and excellent electrical conductivity.^[^ ^23^, ^24^, ^25^ ^]^ Research efforts to form Li metal on Cu CCs for use in LMBs are ongoing: however, the use of Cu often results in the growth of Li dendrites owing to its strong lithiophobicity, leading to uneven Li deposition.^[^ ^26^, ^27^ ^]^ Various surface‐treatment techniques have been developed to address this issue. In particular, the application of lithiophilic materials such as ZnO, Ag, NiO, or graphene to Cu foil promotes a more uniform Li^+^ flux, thereby enabling more even Li deposition.^[^ ^28^, ^29^, ^30^ ^]^ Notably, ZnO induces the formation of a Li_2_O‐based SEI during lithiation and facilitates the development of a LiF‐rich SEI layer with enhanced Li^+^ diffusion. Furthermore, ZnO is a promising candidate for Li metal anodes owing to its lithiophilicity, which promotes uniform Li nucleation and improves wettability with molten Li.^[^ ^31^, ^32^, ^33^ ^]^

Herein, we introduce a novel and industrially applicable strategy for the fabrication of ZnO/ Zn(OH)_2_ nanosheets on Cu foil (denoted ZOH NSs–Cu foil) via an electroconversion reaction. Cu(OH)_2_ nanowires were initially formed on Cu foil (denoted Cu(OH)_2_ NWs–Cu foil) via anodization. The subsequent reaction of the Cu(OH)_2_ NWs–Cu foil with Zn^2+^ ions in aqueous 2 m ZnSO_4_ solution forms lithiophilic sites on the surface, thereby transforming Cu(OH)_2_ NWs to ZOH NSs. The lithiophilicity and high electrochemically active surface area of the ZOH NSs–Cu foil resulted in a low nucleation overpotential and lower effective current density, respectively. Furthermore, the formation of a stable SEI layer facilitates Li^+^ ion transport, resulting in low charge‐transfer resistance. The uniform and dense deposition of Li enabled fabrication of a Li@ZOH NSs–Cu foil anode which, when paired with commercial LiFePO_4_ (LFP), achieved superlative capacity retention and ≈100% efficiency over more than 350 cycles at 1 C, even with a low N/P ratio of ≈1.9. This paper presents a scalable electrodeposition strategy for the construction of ZOH NSs on Cu foil, providing an effective approach for stabilizing Li metal anodes with applications in next‐generation high‐energy batteries.

## 2 Results and Discussion

**Scheme 1:**
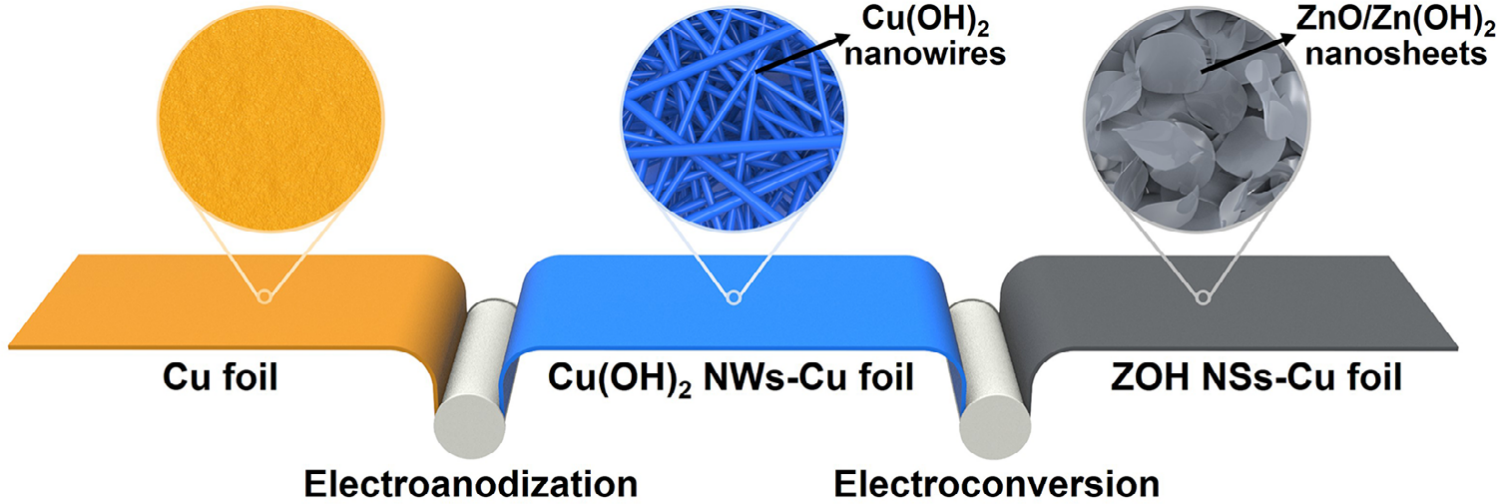
Schematic illustration of the fabrication procedure for ZOH NSs–Cu foil.

The ZOH NSs–Cu foil was fabricated using a facile electrodeposition method that enhanced the number of lithiophilic sites and increased the surface area of the Cu foil, resulting in the formation of ZOH NSs–Cu foil (**Scheme** **1**). The reaction of the surface of the Cu foil with hydroxyl groups during electroanodization in KOH solution resulted in the growth of Cu(OH)_2_ nanowires on the Cu foil surface.^[^ ^34^, ^35^ ^]^ The application of a cathodic current to a Cu(OH)_2_ NWs–Cu foil immersed in a ZnSO_4_ solution induced the electrochemical conversion of Zn^2+^ and Cu^2+^ in the solution, thereby forming a mixed nanosheet structure comprising Zn(OH)_2_ and ZnO on the Cu foil surface.^[^ ^36^ ^]^

**Figure 1:**
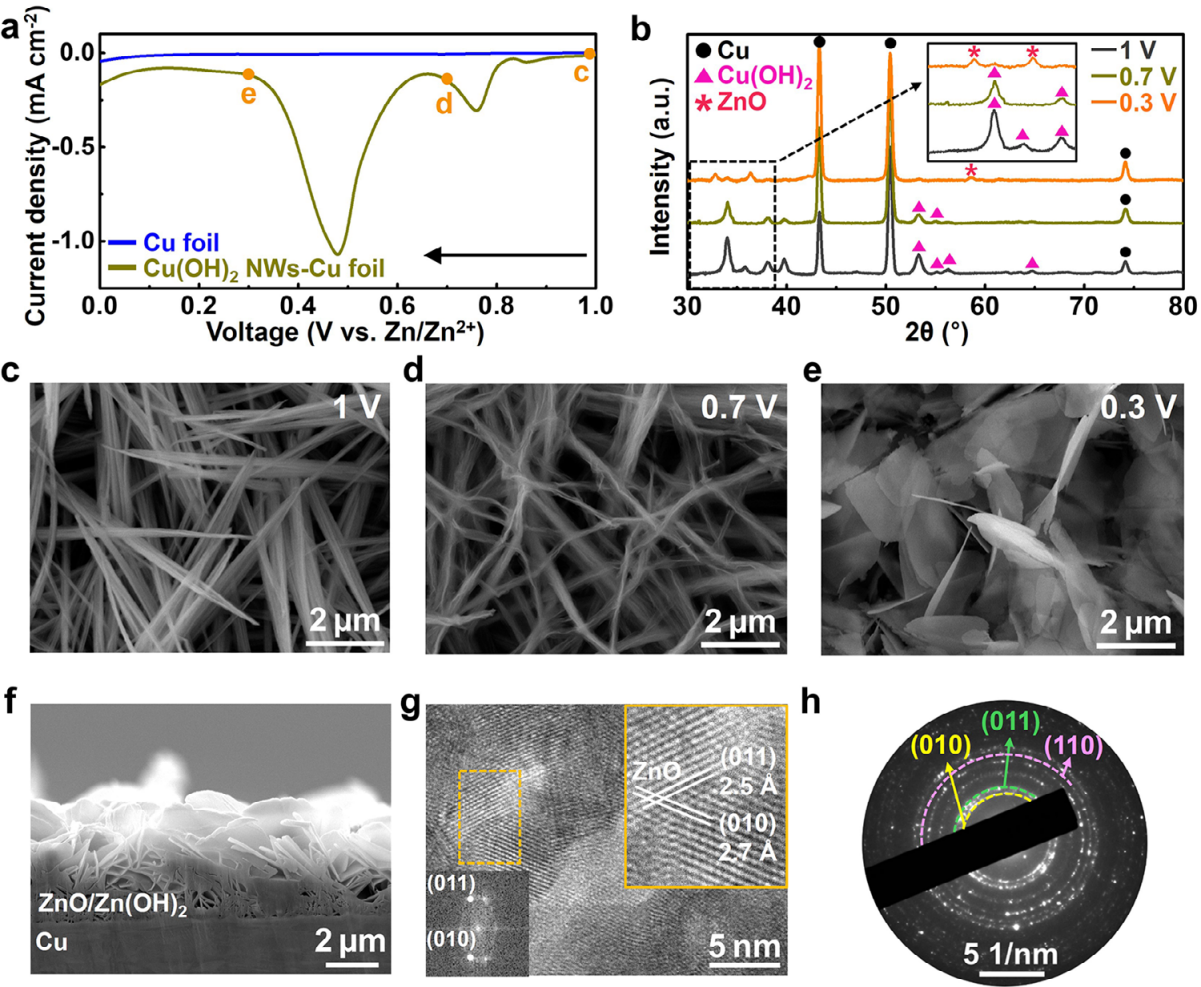
a) LSV curves of Cu foil, and Cu(OH)_2_ NWs–Cu foil during the reaction in ZnSO_4_ solution. b) XRD patterns of Cu(OH)_2_ NWs–Cu foil at 1 V (initial state), 0.7 V, and 0.3 V (post‐reduction). Top‐view SEM images of c) 1 V, d) 0.7 V, and e) 0.3 V. f) Cross‐sectional SEM image of ZOH NSs–Cu foil. g) TEM images of ZOH NSs–Cu foil. The inset shows the corresponding fast Fourier transform (FFT) pattern and inverse FFT of the orange square region. h) Corresponding selected‐area electron diffraction (SAED) pattern.

The mechanism by which the Cu(OH)_2_ NWs–Cu foil was transformed into a ZOH NSs–Cu foil was analyzed using linear sweep voltammetry (LSV), as shown in **Figure** **1**a. To replicate the reaction conditions, Cu(OH)_2_ NWs–Cu foil and Zn metal were immersed in a ZnSO_4_ solution, which was used as an electrolyte in the synthesis of ZOH NSs–Cu foil. The current density was monitored while varying the potential from 1 (open circuit voltage) to 0 V to investigate the electrochemical behavior during the reaction with Zn^2+^ ions. The current density of the Cu foil exhibited minimal changes within this potential window: however, the voltammogram of the Cu(OH)_2_ NWs–Cu foil showed two distinct reduction peaks at ≈0.76 V and 0.48 V, thereby confirming the reaction between the Cu^2+^ ions in the Cu(OH)_2_ NWs–Cu foil and the Zn^2+^ ions in the solution. Furthermore, X‐ray diffraction (XRD) patterns obtained at 1 V (initial state), 0.7, and 0.3 V (post‐reduction) identified the compositional changes that occurred during the reduction process. At 1 V, before the reaction occurred, only peaks corresponding to the Cu foil and Cu(OH)_2_ were observed (Figure 1b). In contrast, as the initial reduction proceeded at 0.7 V, the intensity of the Cu(OH)_2_ peaks decreased significantly, with some disappearing entirely, and no new peaks were detected. This suggests that the Cu^2+^ in the Cu(OH)_2_ NWs–Cu foil partially reacted with Zn^2+^ in solution, leading to the conversion of Cu(OH)_2_ into Zn(OH)_2_ and the reduction of Cu^2+^ to metallic Cu (Equation 1). However, the amount of Zn(OH)_2_ formed is lower than the detection limit of the XRD spectrometer. At 0.3 V, the reduction reaction was complete, resulting in the disappearance of the remaining Cu(OH)_2_ and the emergence of a new ZnO peak. The ZnO phase may be derived from the complete reaction of the remaining Cu^2+^ in Cu(OH)_2_ with Zn^2+^ as the reduction time increases (Equation 2).

(1) $$\mathrm{Cu}{\left(\mathrm{OH}\right)}_{2}+{\mathrm{Zn}}^{2+}+{2e}^{-}\to \mathrm{Cu}+\mathrm{Zn}{\left(\mathrm{OH}\right)}_{2}$$

(2) $$\mathrm{Cu}{\left(\mathrm{OH}\right)}_{2}+{\mathrm{Zn}}^{2+}+2e^{-}\to \mathrm{Cu}+\mathrm{ZnO}+H_{2}O$$

The morphological evolution and phase changes in the electrodes during the synthesis were further confirmed using scanning electron microscopy (SEM) and energy‐dispersive X‐ray spectroscopy (EDS) mapping. Cu(OH)_2_ NWs were uniformly dispersed on the surface of the Cu foil during anodization (Figure 1c). The morphological changes corresponding to the reduction potential of the ZnSO_4_ solution were also observed. At 0.7 V, Cu(OH)_2_ was partially converted to Zn(OH)_2_, with the corresponding reduction of Cu^2+^ to metallic Cu, causing the nanowires to bend and become thinner (Figure 1d). As the reduction reaction progressed, the remaining Cu(OH)_2_ was fully converted to ZnO, forming a nanosheet structure with a dark gray surface (Figure 1e,f and Figure S1, Supporting Information). The EDS maps and corresponding atomic composition at different voltages during the conversion reaction confirmed the successful transformation of Cu(OH)_2_ to Zn(OH)_2_ and ZnO (Figure S2, Supporting Information). Notably, at 0.7 V, where the first reduction peak was complete, the O/(Cu+Zn) atomic composition (at.%) ratio was essentially identical to that at 1 V, suggesting that the reduction peak observed at 0.7 V originates from the reduction of Cu^2+^ during the formation of Zn(OH)_2_. In contrast, at 0.3 V, where ZnO is formed, the O/(Cu+Zn) atomic composition ratio decreases significantly, which was attributed to a decrease in the oxygen content during the conversion of Cu(OH)_2_ to ZnO. The transmission electron microscopy (TEM) image of the sample subjected to LSV at up to 0.3 V (Figure 1g) shows lattice spacings of 2.5 and 2.7 Å, which correspond to the (011) and (010) planes of ZnO, respectively. Additionally, the selected‐area electron diffraction patterns (Figure 1h) display the (010), (011), and (110) planes of ZnO. Furthermore, as shown in Figure S3 (Supporting Information), a lattice spacing of 1.7 Å associated with the (213) plane of Zn(OH)_2_ is observed,^[^ ^36^ ^]^ indicating the successful conversion of Cu(OH)_2_ to Zn(OH)_2_ and ZnO.

**Figure 2:**
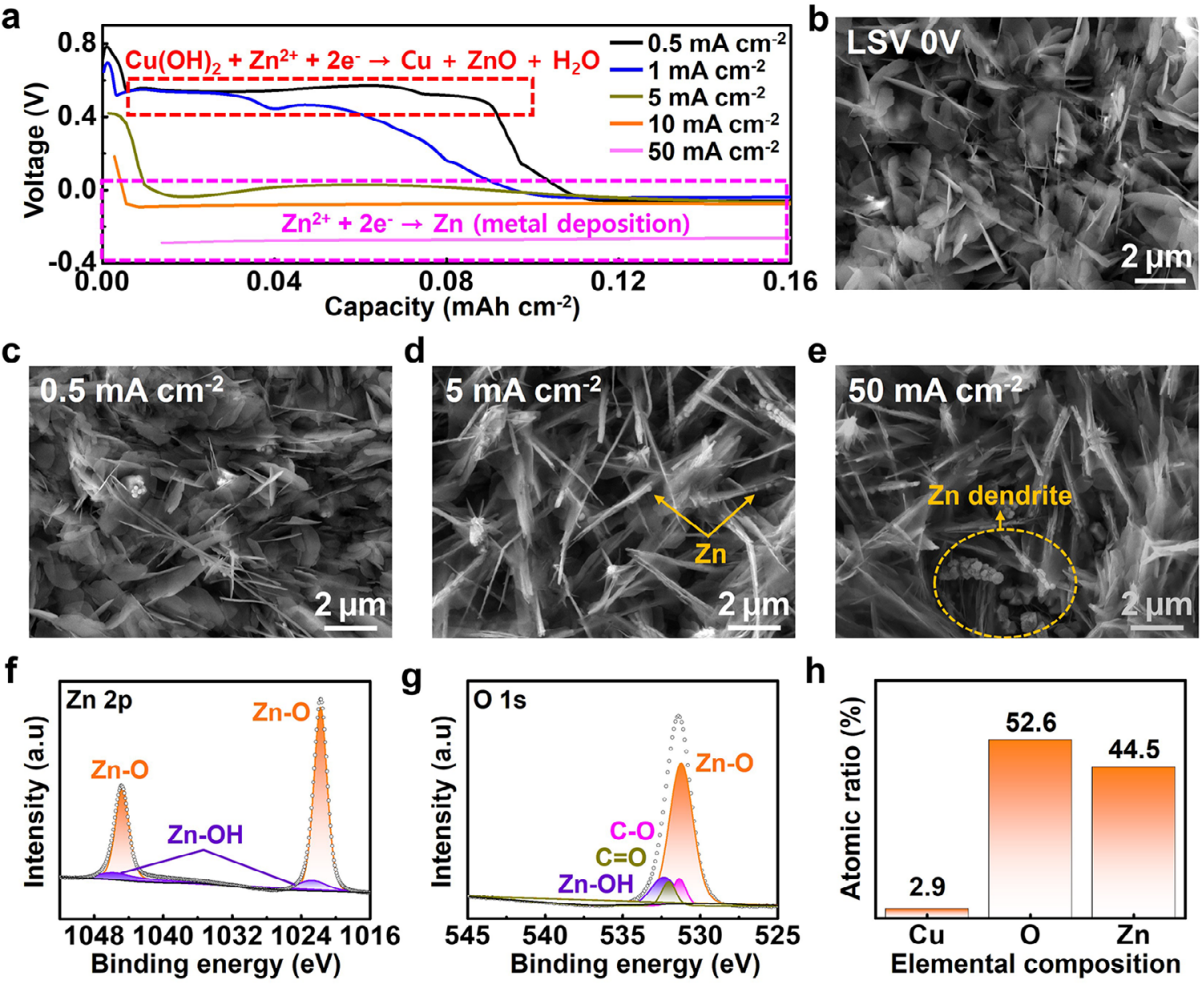
a) Voltage‐capacity curves of Cu(OH)_2_ NWs–Cu foil at different current densities in ZnSO_4_ solution. b) Top‐view SEM image of ZOH NSs–Cu foil obtained through the LSV method. c–e) Surface evolution at different current densities during the electroconversion of Cu(OH)_2_ NWs–Cu foil to ZOH NSs–Cu foil. XPS Spectra of ZOH NSs–Cu foil: f) Zn 2p and g) O 1s. h) Atomic ratios of Cu, O, and Zn in ZOH NSs–Cu foil.

The use of continuous processes in the fabrication of large‐area electrodes is critical for the commercialization of battery systems. The galvanostatic method is easily applicable in large‐scale industrial electrochemical synthesis:^[^ ^37^ ^]^ thus, in this study, the conditions were optimized by adjusting the current density to enable the fabrication of large‐area electrodes via the galvanostatic method. Under galvanostatic conditions, the voltage of the Cu foil remained essentially unchanged regardless of the applied current density (Figure S4, Supporting Information). In contrast, the voltage behavior of the Cu(OH)_2_ NWs–Cu foil was dependent on the current density (**Figure** **2**a). At current densities greater than 5 mA cm^−2^, overpotential‐induced voltage changes were observed, and no plateau was observed: however, at lower current densities of 0.5 and 1 mA cm^−2^, a plateau appeared at ≈0.5 V, corresponding to the voltage at which the conversion reaction occurs, as confirmed by LSV (Figure 1a). Figure 2b shows an SEM image obtained during the reaction of Cu(OH)_2_ NWs–Cu foil with Zn^2+^ ions up to 0 V using LSV, a potentiodynamic method. Figure 2c–e and Figure S5 (Supporting Information) show the surface evolution of the samples synthesized at different current densities via the galvanostatic method. At low current densities of 0.5 and 1 mA cm^−2^, a plateau was observed, and ZnO and Zn(OH)_2_ nanosheets were formed via the conversion reaction. Increasing the current density above 5 mA cm^−2^ inhibited the conversion of Cu(OH)_2_ to ZnO, resulting in the deposition of Zn metal along the Cu(OH)_2_ nanowire and the eventual precipitation of the Cu(OH)_2_ nanowires. The optimal reaction conditions therefore include a current density of 0.5 mA cm^−2^, at which the formation of ZnO was maximized while Zn metal deposition was suppressed. This result confirms that the surface obtained using LSV (Figure 2b) is consistent with that obtained using the galvanostatic method, demonstrating the reproducible and scalable galvanostatic synthesis of the ZOH NSs–Cu foil. The chemical composition of the ZOH NSs–Cu foil was further analyzed by X‐ray photoelectron spectroscopy (XPS) following completion of the reaction (Figure 2f,g). The Zn 2p spectrum shows two peaks characteristic of ZnO peaks at 1021.8 eV (Zn 2p_1/2_) and 1044.8 eV (Zn 2p_3/2_), indicating that ZnO was the dominant phase.^[^ ^38^, ^39^ ^]^ Less intense peaks related to Zn–OH bonds were also observed, demonstrating the partial conversion of Cu(OH)_2_ to Zn(OH)_2_.^[^ ^40^ ^]^ In the O 1s spectrum, while minor C─O and C═O peaks were observed due to atmospheric exposure, the dominant presence of Zn–O bonds clearly confirms the successful conversion of the Cu(OH)_2_ NWs–Cu foil to the ZOH NSs–Cu foil. The observed elemental composition further supports the conclusion that the primary phase formed during the reaction is the ZnO (Figure 2h).

**Figure 3:**
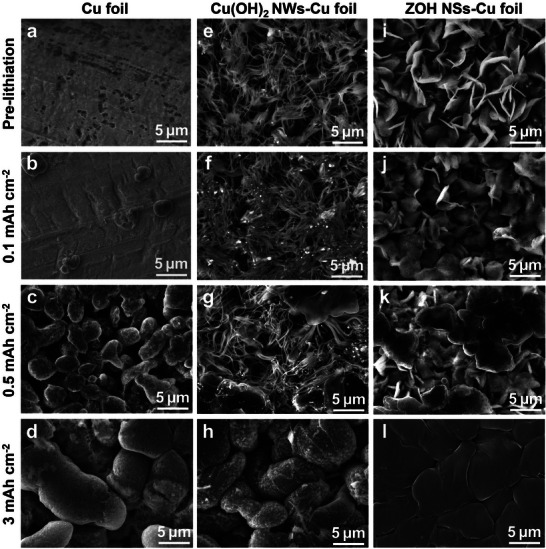
Top‐view SEM images of Cu foil, Cu(OH)_2_ NWs–Cu foil, and ZOH NSs–Cu foil after pre‐lithiation and Li deposition. (a,e,i) Pre‐lithiation, (b,f,j) 0.1 mAh cm^−2^, (c,g,k) 0.5 mAh cm^−2^, and (d,h,l) 3 mAh cm^−2^.

The surface morphology was observed while incrementally increasing the Li deposition capacity to analyze the influence of the CC on the deposition behavior. During the initial stages, an uneven surface was formed the amount of deposited Li increased owing to the random nucleation of Li on the Cu foil (**Figure** **3**a–d). Similarly, localized nucleation occurred on the Cu(OH)_2_ NWs–Cu foil during the initial stages, leaving parts of the surface exposed and resulting in a nonuniform surface morphology even after Li deposition at 3 mAh cm^−2^ (Figure 3e–h). In contrast, the ZOH NSs–Cu foil underwent a pre‐lithiation process involving alloying reactions between Zn and Li, thereby thickening the nanosheets (Figure 3i). Accordingly, the surface was uniformly covered as Li deposition increased, forming a dense and smooth structure (Figure 3j–l). The structural stability of the Li metal anode enabled by the ZOH NSs layer was further evaluated after 50 cycles at 2 mA cm^−2^ with 1 mAh cm^−2^ capacity. As shown in Figure S6 (Supporting Information), Cu foil and Cu(OH)_2_ NWs–Cu foil exhibited nonuniform Li deposition with severe Li dendrites and dead Li, resulting in significant thickness increases above 30 µm. In contrast, the ZOH NSs–Cu foil maintained uniform and compact Li deposition without dendrite formation. The cross‐sectional SEM image further confirmed that the ZOH NSs layer can accommodate repetitive Li plating/stripping with minimal volume change.

**Figure 4:**
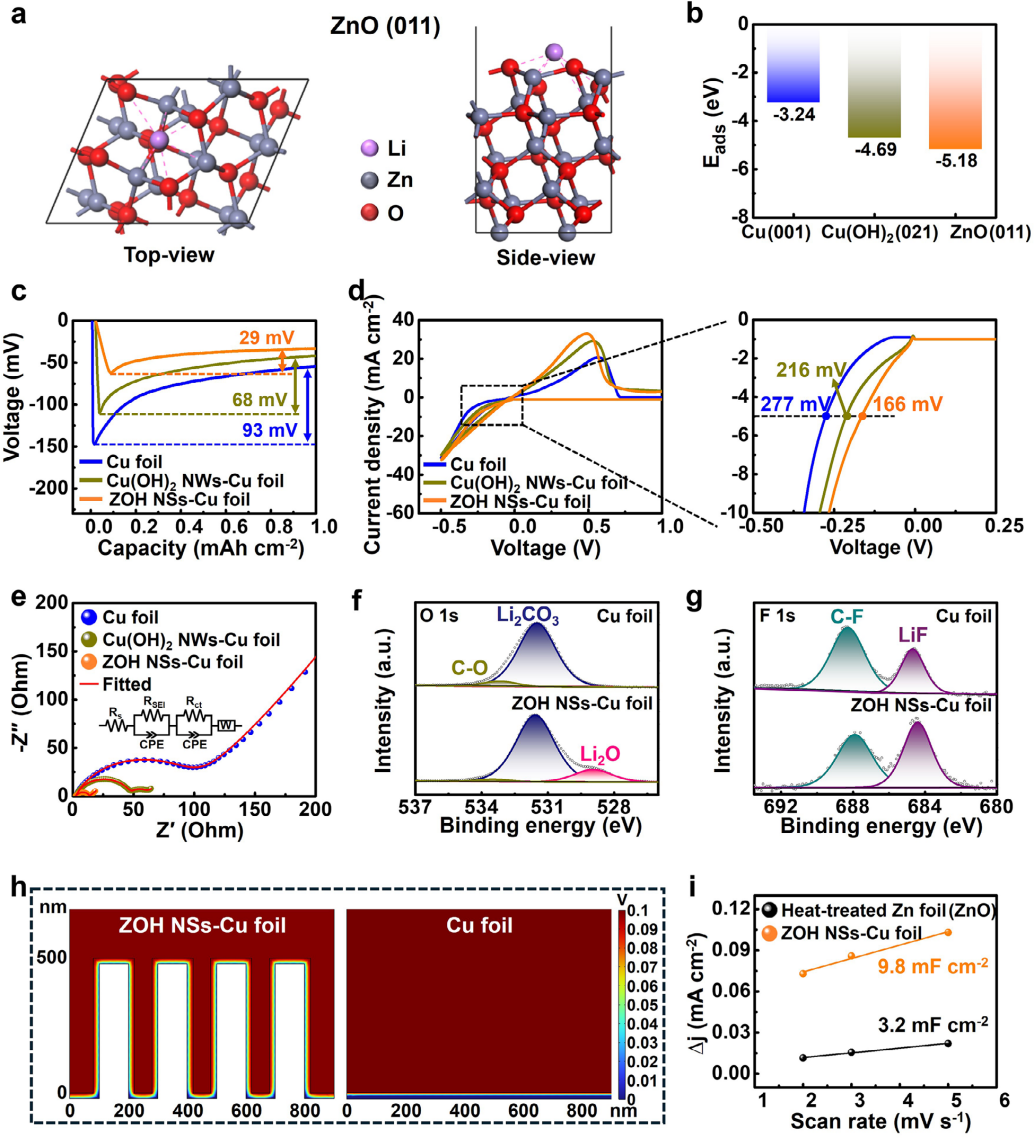
a) Top and side views of the Li adsorption configurations on ZnO (011). b) Comparison of adsorption energy of a Li atom with Cu (001), Cu(OH)_2_ (021), and ZnO (011). c) Li nucleation overpotential on various current collectors at current density of 1 mA cm^−2^. d) CV curves of asymmetric cells with Cu foil, Cu(OH)_2_ NWs–Cu foil, and ZOH NSs–Cu foil at a scan rate of 10 mV s^−1^. e) Nyquist plots of Cu foil, Cu(OH)_2_ NWs–Cu foil, and ZOH NSs–Cu foil. XPS spectra of Cu foil and ZOH NSs–Cu foil: f) O 1s and g) F 1s. h) COMSOL simulations of Li deposition on the Cu foil and ZOH NSs–Cu foil: potential distribution. i) ECSA of the heat‐treated Zn foil (ZnO) and ZOH NSs–Cu foil.

The mechanism underlying the enhanced Li deposition behavior of the ZOH NSs–Cu foil was investigated using a combination of computational and analytical approaches. The adsorption energy (*E*_ads_) of each CC was simulated using density functional theory calculations to compare the affinity of the various CCs for Li. **Figure** **4**a shows the main crystallographic plane of the ZOH NSs–Cu foil. The *E*_ads_ values of Li on Cu foil, Cu(OH)_2_ NWs–Cu foil, and ZOH NSs–Cu foil were –3.24, –4.69, and –5.18 eV, respectively, indicating that Li adsorption is most favorable on the surface of the ZOH NSs–Cu foil, which has the highest *E*_ads_ value (Figure 4b and Figure S7, Supporting Information).^[^ ^41^, ^42^ ^]^ Plots of the initial voltage curves during Li deposition (Figure 4c) revealed that the ZOH NSs–Cu foil exhibited a significantly lower nucleation overpotential (29 mV) than the Cu foil (93 mV) or Cu(OH)_2_ NWs–Cu foil (68 mV), suggesting that the superior lithiophilicity of the ZOH NSs–Cu foil facilitated Li nucleation and uniform Li deposition across the electrode surface. The Li plating/stripping behavior of ZOH NSs–Cu foil was further analyzed using cyclic voltammetry (CV) within a potential range of –0.5 to 1 V at a scan rate of 10 mV s^−1^ (Figure 4d). At a current density of –5 mA cm^−2^, the ZOH NSs–Cu foil exhibited the lowest Li plating overpotential (166 mV) among the examined CCs, indicating that the abundant lithiophilic sites on ZOH NSs–Cu foil improve the charge transfer efficiency, thereby enhancing the overall reaction kinetics.^[^ ^43^, ^44^ ^]^ Although the DFT calculations were performed under vacuum at 0 K, which differs from the actual electrolyte environment at 298 K where Li reactions occur, the results indicate that Li adsorption on the ZOH NSs surface leads to a more thermodynamically stable state. This suggests that the surface exhibits lithiophilic characteristics. Such a tendency is consistent with experimental observations, where Li nucleation occurs at lower potentials and the overpotential is reduced under identical current densities.^[^ ^45^ ^]^ The interfacial resistance of each CC during lithiation was determined using electrochemical impedance spectroscopy with asymmetric cells. The ZOH NSs–Cu foil exhibited the lowest interfacial resistance after lithiation (Figure 4e). After 100 cycles, the ZOH NSs–Cu foil still maintained the lowest interfacial resistance (Figure S8, Supporting Information). To further verify the interfacial reaction kinetics depending on the CC, EIS measurements were performed using symmetric cells (Figure S9, Supporting Information). The Li@ZOH NSs–Cu foil electrode exhibited the lowest SEI impedance, which is consistent with the trend observed in Figure 4e. After 50 cycles, although all symmetric cells exhibited reduced impedance due to the activation process, the Li@ZOH NSs–Cu foil still maintained the lowest SEI resistance. These results support that the ZOH NSs layer facilitates the formation of a stable SEI, thereby enhancing Li⁺ transport at the electrode–electrolyte interface during prolonged cycling.^[^ ^46^, ^47^ ^]^ Moreover, the ZOH NSs–Cu foil exhibits a higher exchange current density than the other CCs, further demonstrating its improved charge‐transfer kinetics (Figure S10, Supporting Information).

XPS was conducted to better understand the contribution of the SEI layer to the electrochemical properties of the ZOH NSs–Cu foil. The C 1s and O 1s spectra (Figure 4f and Figure S11a,b, Supporting Information) reveal the presence of carbonaceous species, including C–O and Li_2_CO_3_ originating from irreversible side reactions such as the decomposition of organic solvents in the electrolyte.^[^ ^21^, ^48^, ^49^ ^]^ The F 1s spectrum of the ZOH NSs–Cu foil shows an intense peak attributed to LiF, suggesting that ZOH NSs induce the decomposition of LiTFSI, resulting in the in situ formation of LiF, which exhibits excellent ionic conductivity (Figure 4g).^[^ ^49^, ^50^ ^]^ In the S 2p spectra (Figure S11c,d, Supporting Information), a distinct Li_2_S peak is observed for the ZOH NSs–Cu foil but not for the bare Cu foil, further supporting that the ZOH NSs promote LiTFSI decomposition. Additionally, the formation of Li_3_N was detected in both Cu foil and ZOH NSs–Cu foil, likely arising from the reductive decomposition of the LiNO_3_ additive (Figure S11e,f, Supporting Information). To further analyze the differences in Li deposition behavior across different CCs (Figure 4h), Li deposition simulations were conducted using COMSOL software, based on previous electrochemical analyses. The modeled Cu foil electrode–electrolyte interface exhibited abrupt voltage changes due to its low ionic conductivity and high charge transfer resistance. Similarly, the Cu(OH)_2_ NWs–Cu foil demonstrated increased potential gradients at the electrode‐electrolyte interface, attributed to relatively high SEI resistance and uneven ion concentration gradients (Figure S12, Supporting Information).^[^ ^51^ ^]^ In contrast, the superior interfacial properties of the ZOH NSs–Cu foil effectively reduced the potential gradient at the interface, leading to improved Li deposition characteristics. Furthermore, the Brunauer–Emmett–Teller (BET) surface area analysis results of the ZOH NSs–Cu foil are shown in Figure S13 (Supporting Information). Although the absolute surface area value of the ZOH NSs–Cu foil was relatively low (0.50 m^2^ g^−1^) due to the extremely thin ZOH NSs layer deposited on the 18 µm‐thick Cu foil, it represents a significant increase compared to the negligible surface area of the bare Cu foil (0.03 m^2^ g^−1^). This increase indicates substantial morphological changes induced by the formation of nanosheets. This morphological change is expected to enhance the electrochemically active surface area. To elucidate the structural advantages of the ZOH NSs–Cu foil, its electrochemical surface area (ECSA) was evaluated. Specifically, the ECSA of ZnO, the primary component of the ZOH NSs–Cu foil, was compared with that of heat‐treated Zn foil (ZnO) at 300 °C (Figure S14, Supporting Information). As shown in Figure 4i and Figure S15 (Supporting Information), the double‐layer capacitance (C_dl_) value of the ZOH NSs–Cu foil was determined to be 9.8 mF cm^−2^, approximately three times higher than that of the heat‐treated 2D Zn foil (3.2 mF cm^−2^). This substantial increase in the areal surface area demonstrates that the 3D architecture of the ZOH NSs–Cu foil provides a greater number of active sites per unit area, thereby reducing the local current density.^[^ ^52^, ^53^ ^]^

**Figure 5:**
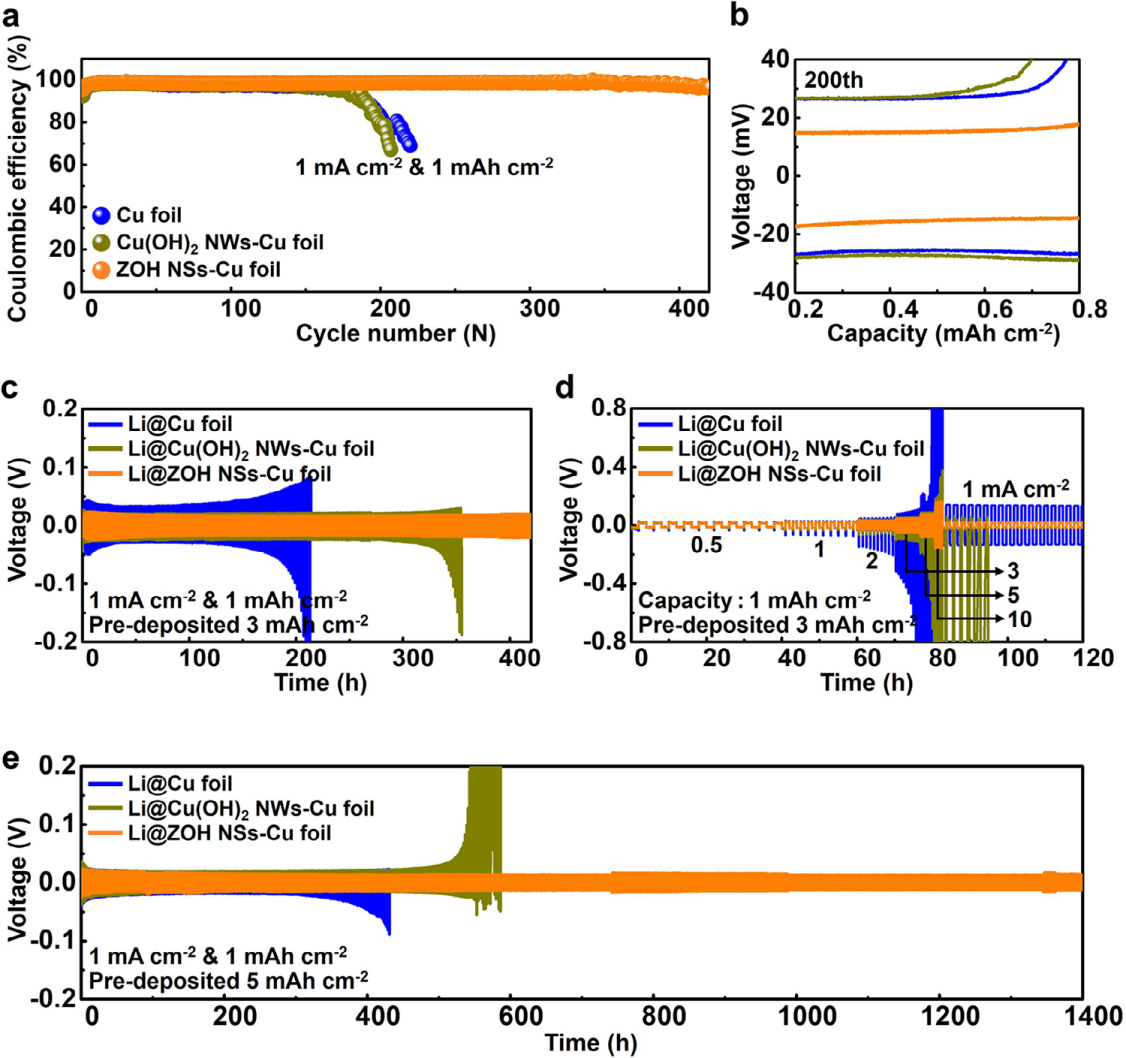
Electrochemical performance of asymmetric and symmetric cells with different current collectors. a) CEs of asymmetric cells with Cu foil, Cu(OH)_2_ NWs–Cu foil, and ZOH NSs–Cu foil at a current density of 1 mA cm^−2^ with a capacity of 1 mAh cm^−2^. b) Voltage–capacity curves at a current density of 1 mA cm^−2^ with a capacity of 1 mAh cm^−2^ at the 200^th^ cycle. c) Voltage–time profiles of symmetric cells with Li@Cu foil, Li@Cu(OH)_2_ NWs–Cu foil, and Li@ZOH NSs–Cu foil at a current density of 1 mA cm^−2^ with a capacity of 1 mAh cm^−2^ after Li pre‐deposition of 3 mAh cm^−2^. d) Rate performance of symmetric cells at current densities ranging from 0.5 to 10 mA cm^−2^ with a capacity of 1 mAh cm^−2^. e) Voltage–time profiles at a current density of 1 mA cm^−2^ with a capacity of 1 mAh cm^−2^ after Li pre‐deposition of 5 mAh cm^−2^.

**Figure 6:**
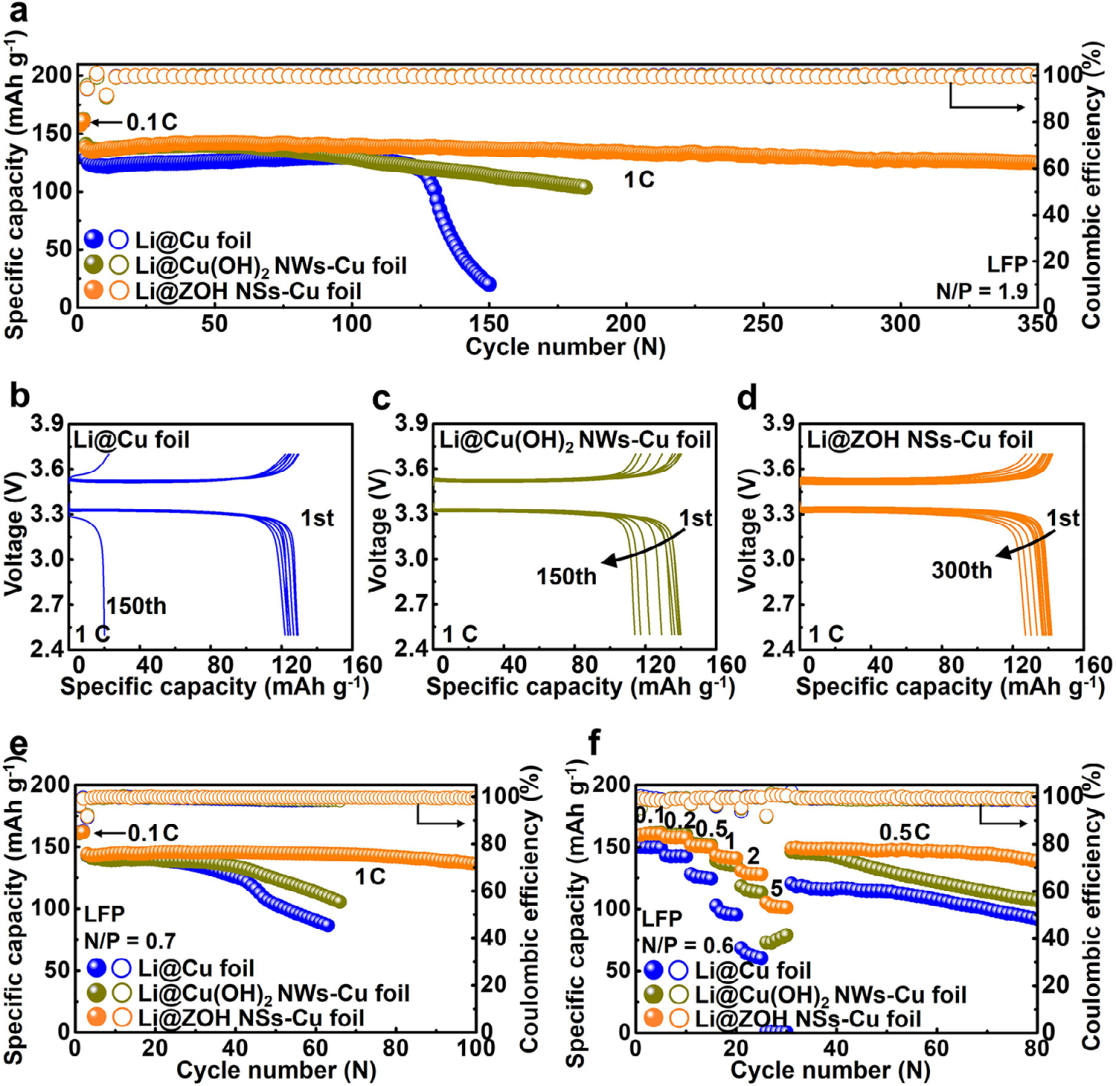
Electrochemical performance of full cells with different anodes paired with the LFP cathode. a) Cycling performance of Li@Cu foil, Li@Cu(OH)_2_ NWs–Cu foil, and Li@ZOH NSs–Cu foil at 1 C (N/P ratio ≈1.9). b–d) Voltage profiles of the three full cells at 1 C. e) Cycling performance of Li@Cu foil, Li@Cu(OH)_2_ NWs–Cu foil, and Li@ZOH NSs–Cu foil at 1 C (N/P ratio ≈0.7). f) Rate performance of full cells with Li@Cu foil, Li@Cu(OH)_2_ NWs–Cu foil, and Li@ZOH NSs–Cu foil.

The influence of the CC on the reversibility of Li plating/stripping was evaluated using asymmetric cells with Cu foil, Cu(OH)_2_ NWs–Cu foil, and ZOH NSs–Cu foil (**Figure** **5**a,b). The ZOH NSs–Cu foil maintained a high Coulombic efficiency (CE) of ≈98.5% over 400 cycles at a capacity and current density of 1 mAh cm^−2^ and 1 mA cm^−2^, respectively, demonstrating superlative stability. In contrast, the CE of Cu foil and Cu(OH)₂ NWs–Cu foil declined sharply after 190 cycles. The ZOH NSs–Cu foil also demonstrated excellent cycling stability at a higher current density of 2 mA cm^−2^, with a high CE retention of ≈98% over 200 cycles (Figure S16, Supporting Information). In contrast, the other CCs exhibited increased polarization due to the continuous growth of Li dendrites during cycling, resulting in rapid capacity fading after 130 cycles. These results demonstrate the high reversibility of the ZOH NSs–Cu foil, which was attributed to the presence of lithiophilic ZnO sites. These sites effectively suppress Li dendrite formation during repeated Li plating/stripping cycles, thereby enhancing cycling stability and extending the lifespan of the CC. The effect of Li deposition on the stabilities of the CCs was assessed using symmetric cells (Figure 5c). The performance of symmetric cells depends on the amount of active Li: thus, the cycling stability was evaluated at different Li deposition levels. At a low Li deposition level (3 mAh cm^−2^), the voltages of the Li@Cu foil and Li@Cu(OH)_2_ NWs–Cu foils fluctuated after 150 and 320 h, respectively, indicating their inability to suppress dead Li formation and Li dendrite growth. In contrast, Li@ZOH NSs–Cu foil exhibited the lowest overpotential of 17 mV and excellent cycling stability for over 450 h. Furthermore, the Li@ZOH NSs–Cu foil consistently exhibited the lowest overpotential across all current densities in rate performance tests conducted at a fixed capacity of 1 mAh cm^−2^ (Figure 5d). Notably, when the current density was reduced from 10 mA cm^−2^ back to 1 mA cm^−2^, the Li@ZOH NSs–Cu foil retained its initial overpotential upon reducing the current density from 10 mA cm^−2^ to 1 mA cm^−2^. Similarly, the Li@ZOH NSs–Cu foil exhibited stable Li plating/stripping behavior when Li deposition was increased to 5 mAh cm^−2^, achieving an exceptional cycling lifespan exceeding 1400 h (Figure 5e and Figure S17, Supporting Information). In contrast, the Cu and Cu(OH)_2_ NWs–Cu foils exhibited voltage fluctuations after 380 and 500 h, respectively, indicating instability. The ZOH NSs–Cu foil promoted the formation of a stable SEI layer rich in Li_2_S, Li_2_O, and LiF while facilitating uniform and dense Li deposition, resulting in low‐voltage hysteresis and enhanced cycling stability.^[^ ^54^, ^55^ ^]^ Similarly, at elevated current density and capacity of 2 mA cm^−2^ and 2 mAh cm^−2^ (Figure S18, Supporting Information), Li@ZOH NSs–Cu foil exhibited a more stable overpotential and longer cycle life compared to the Li@Cu foil and Li@Cu(OH)_2_ NWs–Cu foil, demonstrating the structural and interfacial advantages of the ZOH NSs layer. Notably, Li@ZOH NSs–Cu foil enables a scalable and rapid fabrication process, yielding outstanding electrochemical performance (Table S1, Supporrting Information). The electrochemical performance of the ZOH NSs–Cu foil coupled with a LiFePO_4_ (LFP) cathode was analyzed to demonstrate the practical application of the ZOH NSs–Cu foil as an anode. As shown in **Figure** **6**a, At an N/P ratio of ≈1.9, which is significantly lower than those reported in previous studies (Table S2, Supporting Information), the Li@ZOH NSs–Cu foil delivered a stable CE of ≈100% and an excellent capacity retention exceeding 90% over 350 cycles at 1 C. In contrast, the Li@Cu foil and Li@Cu(OH)_2_ NWs–Cu foils exhibited a significant capacity reduction after 150 cycles, with capacity retentions of ≈15% and 81%, respectively. The capacity‐voltage profiles during cycling at 1 C (Figure 6b–d) revealed that the Li@ZOH NSs–Cu foil electrode achieved the most stable capacity evolution owing to the formation of a stable SEI layer at the interface between the Li@ZOH NSs–Cu foil electrode and the liquid electrolyte. Furthermore, the N/P ratio was reduced to 0.7 through the deposition of a limited Li capacity of 1 mAh cm^−2^ to evaluate the Li@ZOH NSs–Cu foil anode under harsher conditions (Figure 6e). The Li@ZOH NSs–Cu foil maintained a high capacity of 136 mAh g^−1^ even after 100 cycles at 1 C despite this low N/P ratio, whereas the capacities of other electrodes declined sharply after 40 cycles. Additionally, the Li@ZOH NSs–Cu foil demonstrated superior rate performance to the Li@Cu foil and Li@Cu(OH)_2_ NWs–Cu foils (Figure 6f). While the differences in the specific capacities were relatively small at low rates, the performance gap became increasingly pronounced at current densities exceeding 1 C. Notably, the initial capacity of the Li@ZOH NSs–Cu foil remained stable as the current density returned to 0.5 C owing to the enhanced Li^+^ ionic conductivity of the LiF‐rich SEI layer and the reduced local current density at high rates arising from the large specific surface area of the 3D nanosheets. Considering the N/P ratio, the performance of the Li@ZOH NSs–Cu foil full cell surpassed that of existing cells (Table S2, Supporting Information). These findings confirm the applicability of ZOH NSs–Cu foil as an anode for high‐energy‐density Li metal batteries.

## 3 Conclusion

A novel lithiophilic ZOH NSs–Cu foil was developed using a scalable electrodeposition method, offering a robust strategy for stabilizing Li metal anodes. The ZOH NSs effectively reduced the Li nucleation overpotential and promoted uniform Li deposition owing to their strong lithiophilicity. Furthermore, their 3D structures provide a high density of electrochemically active sites, thereby lowering the current density. The reduced charge‐transfer resistance and SEI resistance also facilitated Li^+^ ion transport and improved the charge‐transfer kinetics, resulting in dendrite‐free Li growth. The asymmetric cell evaluation of ZOH NSs–Cu foil exhibited excellent stability during repeated Li plating/stripping cycles. Moreover, the symmetric cell demonstrated remarkable cycle stability even under low Li deposition conditions. In a full‐cell configuration with LFP, the Li@ZOH NSs–Cu foil remained stable for over 350 cycles at 1 C with an N/P ratio of 1.9. The simple and scalable electrodeposition approach for the in situ generation of an ion‐conductive SEI layer introduced in this study provides an effective solution for stabilizing Li metal anodes and shows significant potential for advancing the practicality of high‐energy‐density LMBs.

## Conflict of Interest

The authors declare no conflict of interest.

## Supporting information



Supporting Information

## Data Availability

The data that support the findings of this study are available from the corresponding author upon reasonable request.
