# Supplementary material for: Electrodeposited ZnO/Zn(OH)2 Nanosheets as a Functional Interface for Dendrite‐Free Lithium Metal Anodes
Source: Small. 2025 May 30;21(31):2503607. doi: 10.1002/smll.202503607 (PMC12332819; doi:10.1002/smll.202503607)
Supplement: Supplementary file 1 — Supporting Information [file SMLL-21-2503607-s001.pdf]

NANO · MICRO
small

Supporting Information

for *Small*, DOI 10.1002/smll.202503607

# Electrodeposited ZnO/Zn(OH)$_2$ Nanosheets as a Functional Interface for Dendrite-Free Lithium Metal Anodes

*Da-Eun Hyun, Jong Chan Choi, Yoon Ho Kim, Yejin Ra, Jae Sol Sim, Jung-Kul Lee* and Yun Chan Kang**

Supporting Information

**Electrodeposited $ZnO/Zn(OH)_2$ Nanosheets as a Functional Interface for Dendrite-Free Lithium Metal Anodes**

*Da-Eun Hyun, Jong Chan Choi, Yoon Ho Kim, Yejin Ra, Jae Sol Sim, Jung-Kul Lee*, Yun Chan Kang**

D.-E. Hyun, J. C. Choi, Y. H. Kim, Y. Ra, J. S. Sim, Prof. Y. C. Kang*
Department of Materials Science and Engineering
Korea university
145 Anam-ro, Seongbuk-gu, Seoul 02841, Republic of Korea
E-mail: yckang@korea.ac.kr

Prof. J.-K. Lee*
Department of Chemical Engineering
Konkuk university
Hwayang-dong, Gwangjin-gu, Seoul 143-701, Republic of Korea
E-mail: jkrhee@konkuk.ac.kr

## Experimental Section

### *Preparation of $Cu(OH)_2$ NWs-Cu foil and ZOH NSs-Cu foil*

A Cu foil fixed on a glass substrate was sequentially rinsed with a 5 wt% sulfuric acid solution and deionized water several times to remove surface impurities. The $Cu(OH)_2$ NWs-Cu foil was synthesized in a one-step via a facile electrodeposition method. In this process, the Cu foil and platinum were employed as the working and counter electrodes, respectively, and immersed in an electrolytic bath containing a 1 M potassium hydroxide (KOH) solution. The electrodes were securely held in place using clamps to ensure stability during deposition. A power supply was used to perform galvanostatic electroanodization (1 mA $cm^{-2}$, 10 min) at room temperature, resulting in the formation of a blue $Cu(OH)_2$ nanowire layer on the surface of the Cu foil (denoted $Cu(OH)_2$ NWs-Cu foil). The $Cu(OH)_2$ NWs-Cu foil was transferred to an electrolytic bath containing a 2 M $ZnSO_4$ solution (pH ≈5), where electroconversion was performed at 0.5 mA $cm^{-2}$ for 20 min using a Zn foil as the counter electrode at room temperature. During this process, the $Cu(OH)_2$ NWs layer on the Cu foil was converted into a mixed layer of ZnO and $Zn(OH)_2$ nanosheets. Finally, the prepared current collectors (CCs) were dried in an oven at 60 °C.

### *Material characterization*

X-ray diffraction (XRD) patterns were collected using a PANalytical diffractometer with Cu Kα radiation (λ=1.5418 Å) to examine the crystallographic properties. The morphologies and structural characteristics of the current collectors (CCs) were examined using field-emission scanning electron microscopy (FE-SEM, TESCAN) equipped with energy-dispersive spectroscopy (EDS) and transmission electron microscopy (TEM). To identify the composition and chemical bonding states of the current collectors (CCs) and the SEI layer, X-ray photoelectron spectroscopy (XPS) analysis was performed using a K-Alpha spectrometer (Thermo Fisher Scientific Inc., USA).

### *Electrochemical characterization*

Electrochemical tests were performed using CR2032-type coin cells assembled in an Ar-filled glove box. Celgard 2400 was used as the separator, and all cells utilized an electrolyte consisting of 1 M lithium bis(trifluoromethanesulfonimide) (LiTFSI) dissolved in a 1:1 volume ratio mixture of 1,2-dimethoxyethane (DME) and 1,3-dioxolane (DOL), with the addition of 2 wt% $LiNO_3$. Electrochemical analyses, including galvanostatic tests, linear sweep voltammetry (LSV), cyclic voltammetry (CV) tests, electrochemical impedance spectroscopy (EIS), and Tafel plot measurements, were conducted using a WonAtech electrochemical workstation. CV tests were carried out within a voltage range of –0.5 to 1 V at a scan rate of 10 mV $s^{-1}$. EIS measurements were performed across a frequency range from 1 MHz to 1 Hz with an amplitude of 10 mV. Tafel plots were obtained from Li||Li symmetric cells scanned at 1 mV $s^{-1}$ over a voltage range of –0.14 to 0.14 V. For the asymmetric cells, galvanostatic cycling tests were performed at current densities of 1 and 2 mA $cm^{-2}$ with a capacity of 1 mAh $cm^{-2}$, and stripping proceeded up to 1 V. For the symmetric cell tests, each CC was first utilized in an asymmetric

cell configuration, where Li was deposited at 3 and 5 mAh $cm^{-2}$. Subsequently, two asymmetric cells with identical Li deposition were disassembled and reassembled to form a symmetric cell. The assembled symmetric cells were then cycled at a current density of 1 mA $cm^{-2}$ with a capacity of 1 mAh $cm^{-2}$. For the full cell tests, $LiFePO_4$ (LFP) electrodes were prepared by casting an *N*-methyl-2-pyrrolidone (NMP)-based slurry onto Al foil. The slurry consisted of LFP powder, Super P carbon black, and polyvinylidene fluoride (PVDF) in a weight ratio of 90:5:5. The LFP mass loading was approximately 8.4–9.4 mg $cm^{-2}$. Prior to cell assembly, all CCs were pre-deposited with Li at 3 and 5 mAh $cm^{-2}$. Galvanostatic charge/discharge tests were performed within a voltage range of 2.5–3.7 V.

***DFT calculation***

Density functional theory (DFT) calculations were performed using the CASTEP module in Materials Studio 2023 (BIOVIA) to investigate the adsorption behavior of Li atoms on different CCs. The generalized gradient approximation (GGA) with the Perdew-Burke-Ernzerhof (PBE) functional was employed to describe exchange-correlation interactions. On-The-Fly Generated (OTFG) ultrasoft pseudopotentials were used with a plane-wave energy cutoff of 500 eV. The adsorption energy ($\Delta E_{ads}$) of on Cu foil, $Cu(OH)_2$, and ZnO surfaces was calculated using the following equation:

$$\Delta E_{ads} = E_{Li+CC} - E_{CC} - E_{Li}$$

where $E_{Li+CC}$ is the total energy of the system with an adsorbed Li atom, $E_{CC}$ is the energy of the current collector, and $E_{Li}$ is the energy of an isolated Li atom. The Brillouin zone was sampled using a 2 × 2 × 1 Monkhorst-Pack k-point grid, and a 15 Å vacuum region was applied to prevent interactions between periodic images. The maximum force convergence criterion was set to 0.01 eV/Å.

***COMSOL Multiphysics simulation***

The Li deposition process was simulated using the Electrodeposition, Tertiary Nernst-Planck model in COMSOL Multiphysics. A 2D cross-sectional model was employed to analyze the deposition behavior. A 20 nm SEI layer was applied to the electrode surface, and its diffusion coefficient and ionic conductivity were derived from EIS measurements. The deposition overpotential was set to –200 mV vs. $Li/Li^+$.

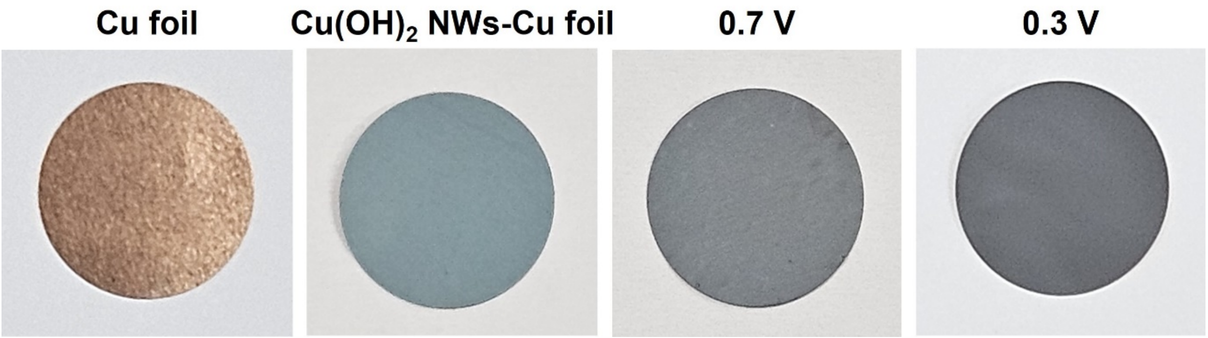


**Figure S1.** Digital photographs of Cu foil, $Cu(OH)_2$ NWs-Cu foil, 0.7 V, and 0.3 V.

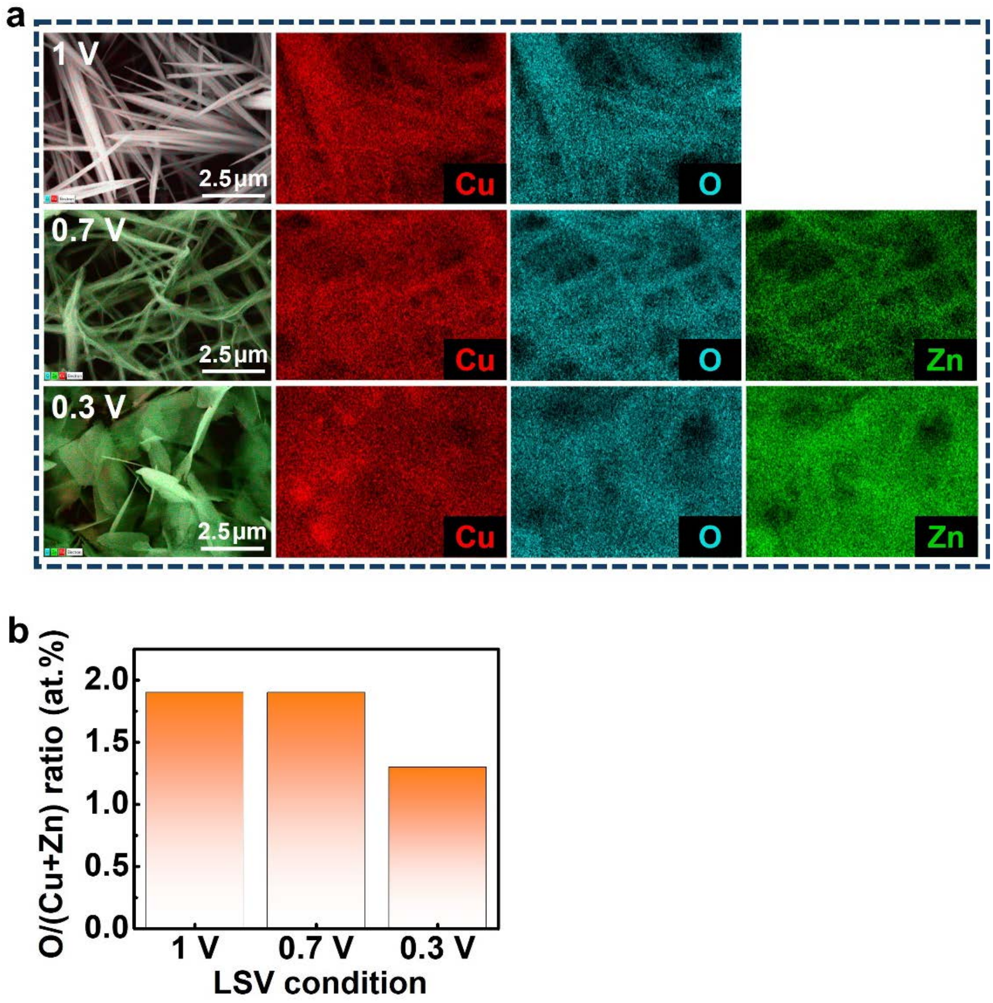


**Figure S2.** a) Surface SEM images and corresponding EDS element mappings at 1 V (initial state), 0.7 V, and 0.3 V (post-reduction). b) Comparison of the O/(Cu+Zn) atomic ratio at 1 V, 0.7 V, and 0.3 V.

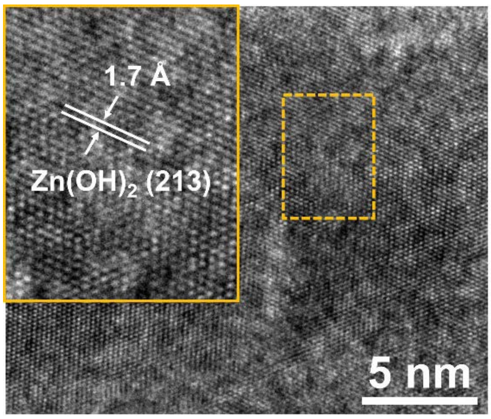


**Figure S3.** TEM images of ZOH NSs-Cu foil. The inset shows the corresponding inverse fast Fourier transform (FFT) of the orange square region.

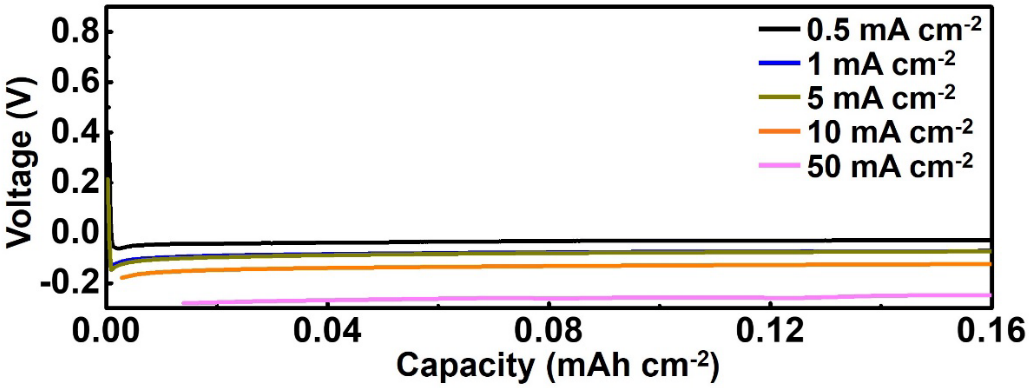


**Figure S4.** Voltage-capacity curves of Cu foil at different current densities in $ZnSO_4$ solution.

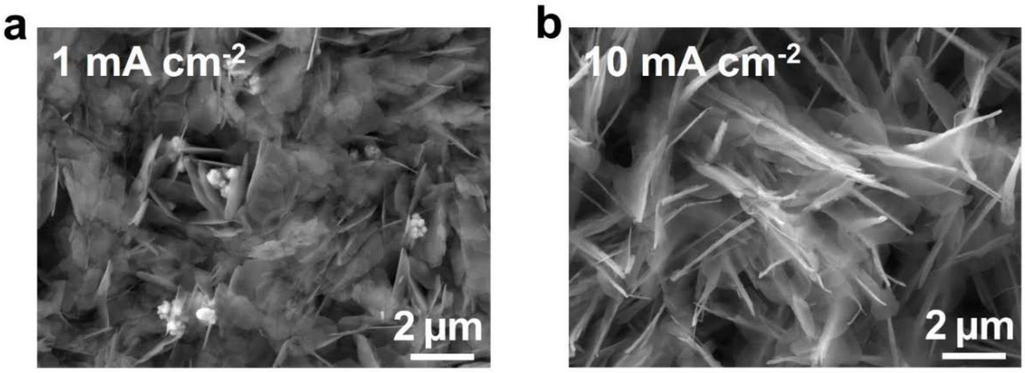


**Figure S5.** Surface evolution at current densities of 1 and 10 mA cm$^{-2}$ during the electrodeposition of $Cu(OH)_2$ NWs–Cu foil to ZOH NSs–Cu foil.

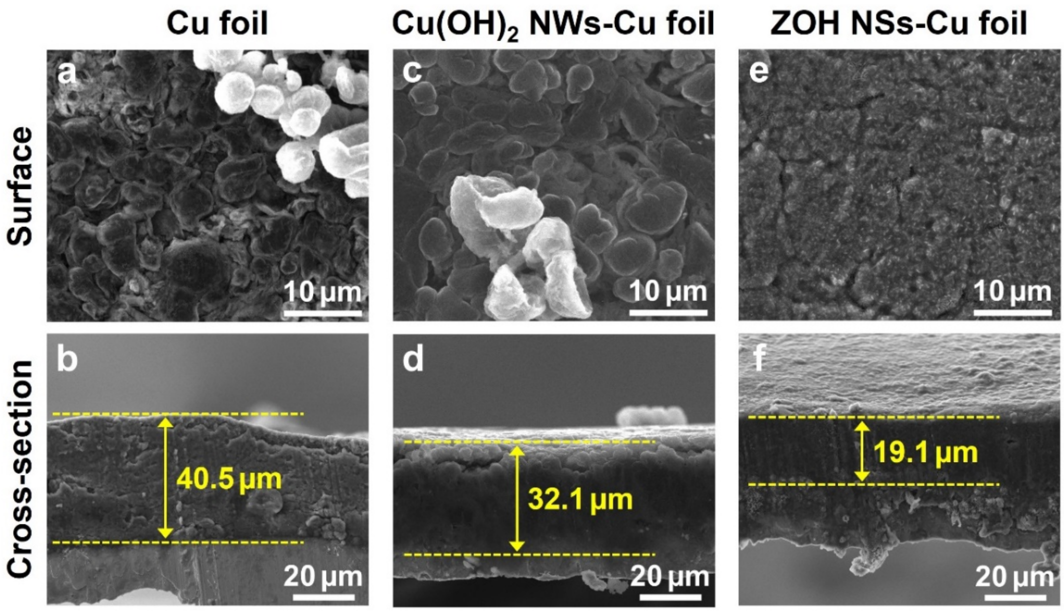


**Figure S6.** Top-view and cross-sectional SEM images of Li-deposited (a,b) Cu foil, (c,d) $Cu(OH)_2$ NWs-Cu foil, and (e,f) ZOH NSs-Cu foil after 50 cycles at a current density of 2 mA $cm^{-2}$ and a capacity of 1 mAh $cm^{-2}$, following Li pre-deposition of 3 mAh $cm^{-2}$.

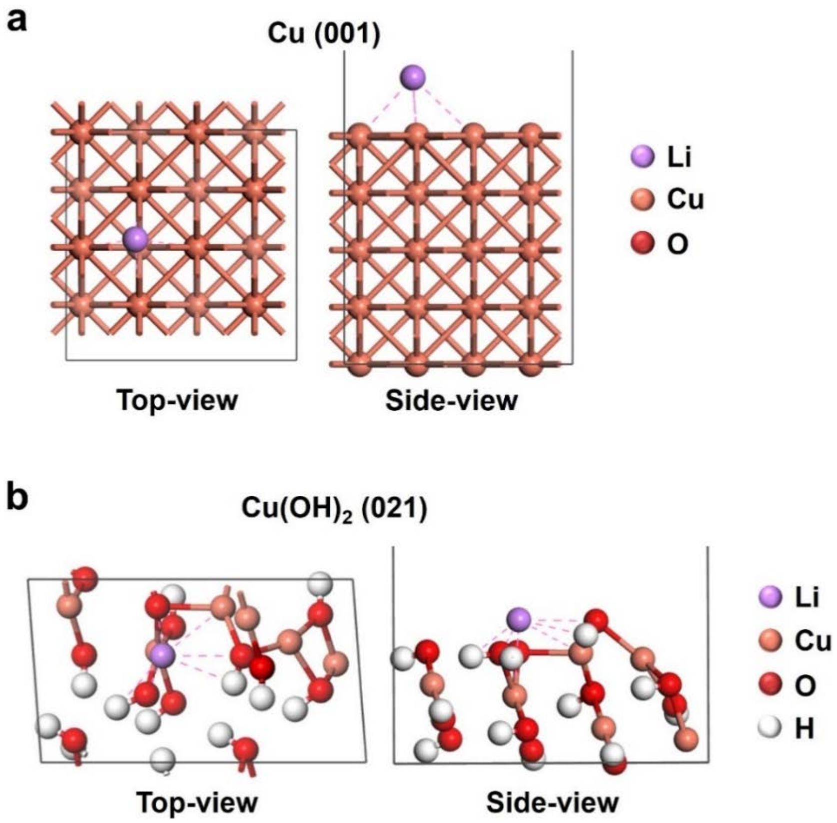


**Figure S7.** Top and side views of the Li adsorption configurations on Cu (001) and $Cu(OH)_2$ (021).

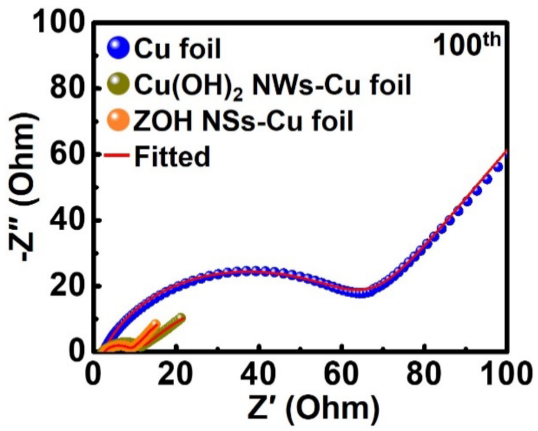


**Figure S8.** Nyquist plots of asymmetric cells using Cu foil, $Cu(OH)_2$ NWs-Cu foil, and ZOH NSs-Cu foil after 100 cycles.

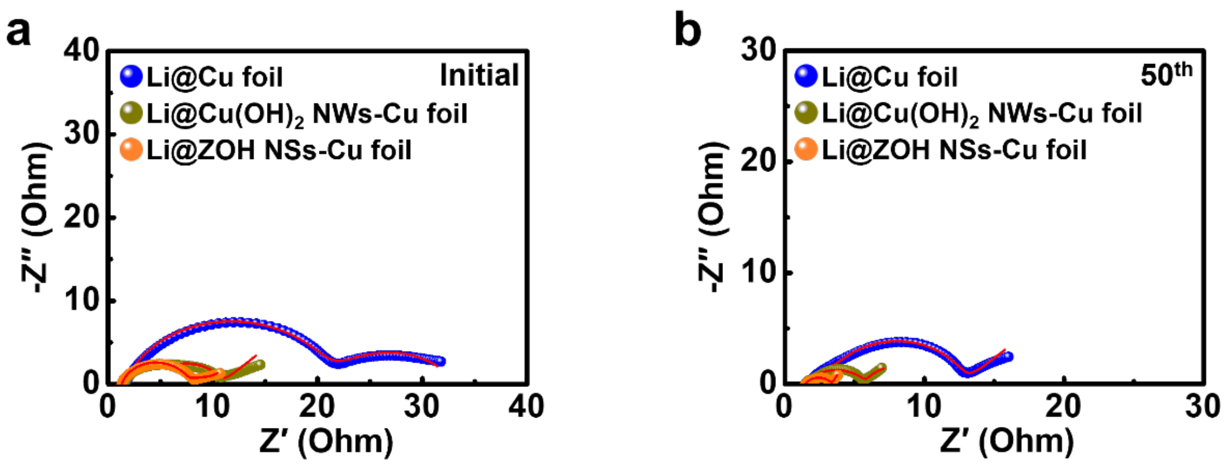


**Figure S9.** Nyquist plots of symmetric cells with Li@Cu foil, Li@$Cu(OH)_2$ NWs-Cu foil, and Li@ZOH NSs-Cu foil at the initial state and after 50 cycles.

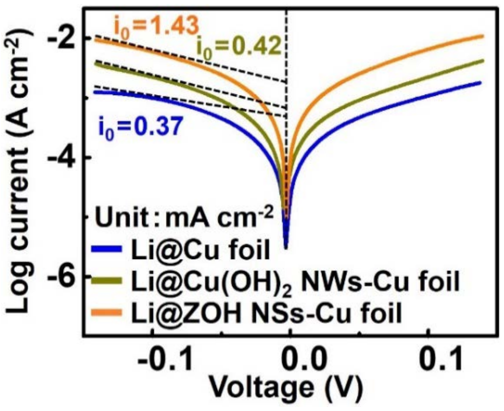


**Figure S10.** Tafel plots of symmetric cells with Li@Cu foil, Li@$Cu(OH)_2$ NWs-Cu foil, and Li@ZOH NSs-Cu foil.

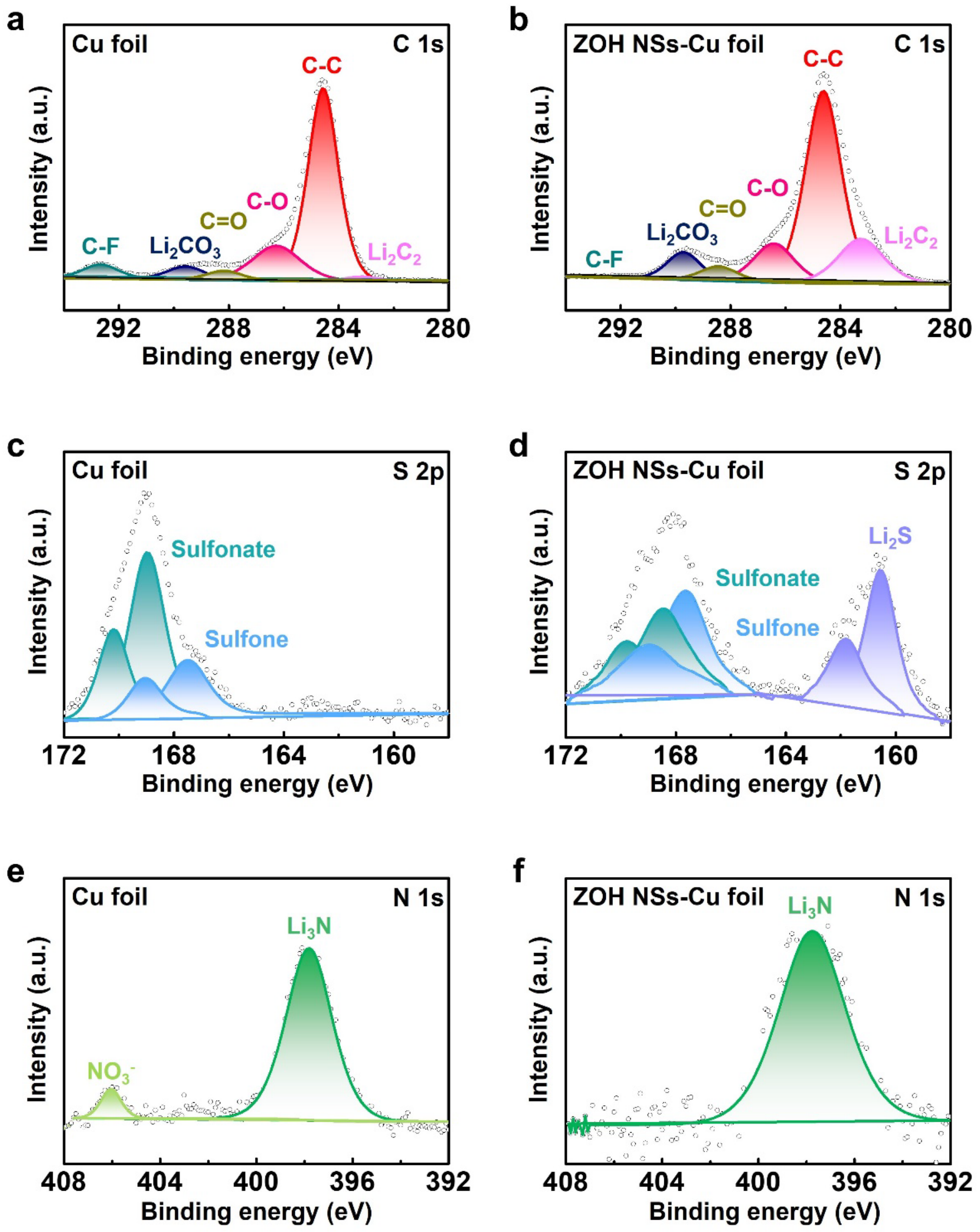


**Figure S11.** XPS spectra of Cu foil and ZOH NSs-Cu foil: a,b) C 1s, c,d) S 2p, and e,f) N 1s regions.

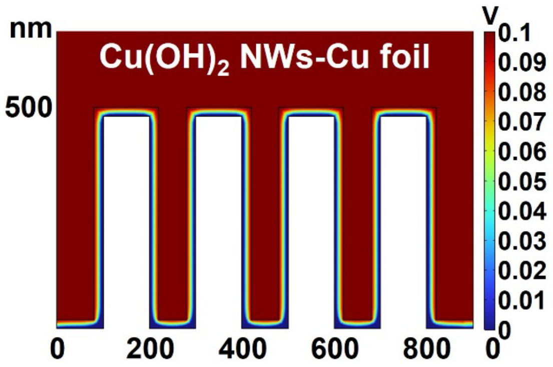


**Figure S12.** COMSOL simulations of Li deposition on the $Cu(OH)_2$ NWs-Cu foil: potential distribution.

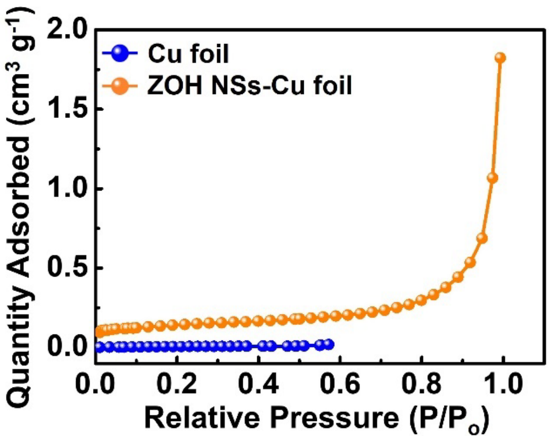


**Figure S13.** Nitrogen adsorption isotherms of Cu foil and ZOH NSs-Cu foil measured for BET surface area analysis.

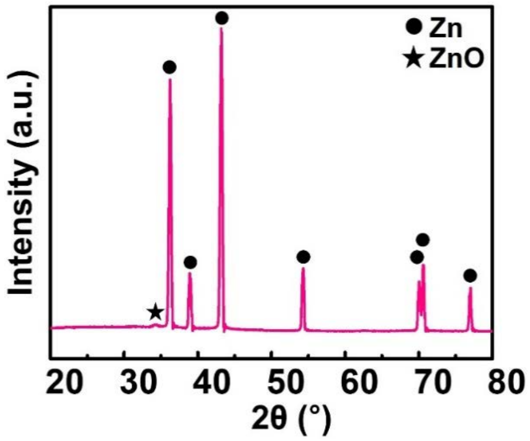


**Figure S14.** XRD pattern of Zn foil obtained by heat treatment at 300 °C.

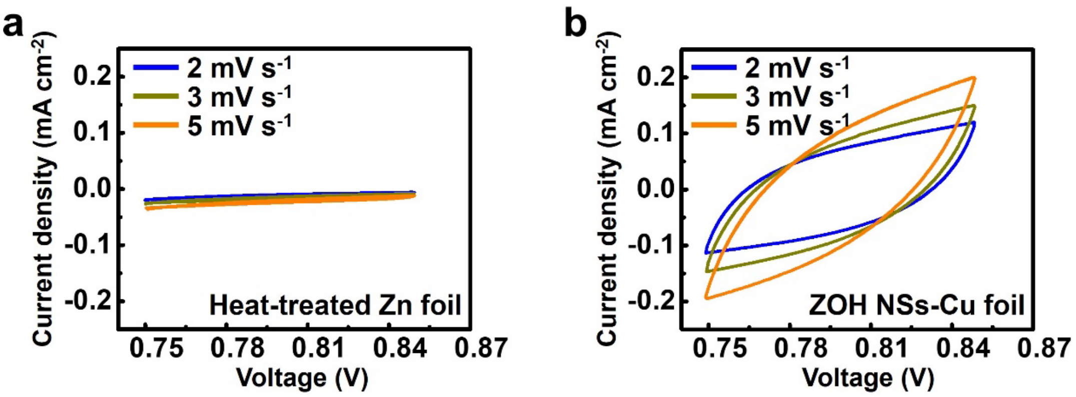


**Figure S15.** CV curves of a) heat-treated Zn foil (ZnO) and b) ZOH NSs-Cu foil at various scan rates ranging from 2 to 5 mV $s^{-1}$.

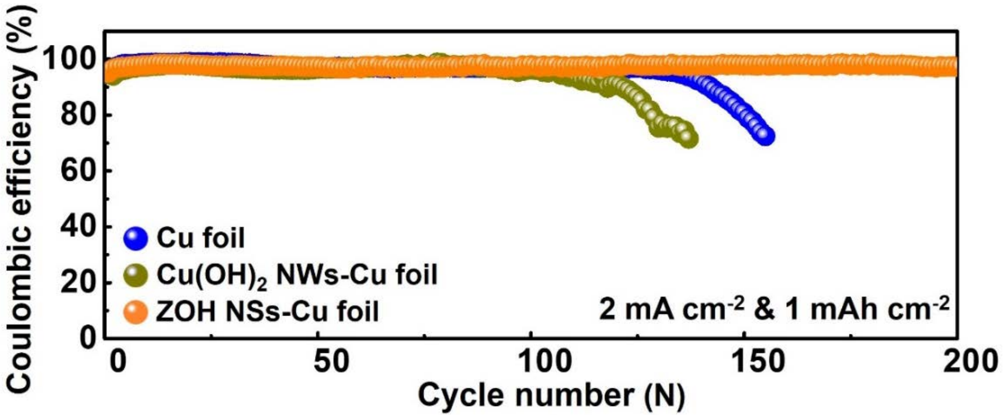


**Figure S16.** CEs of asymmetric cells with Cu foil, $Cu(OH)_2$ NWs-Cu foil, and ZOH NSs-Cu foil at a current density of 2 mA $cm^{-2}$ and a capacity of 1 mAh $cm^{-2}$.

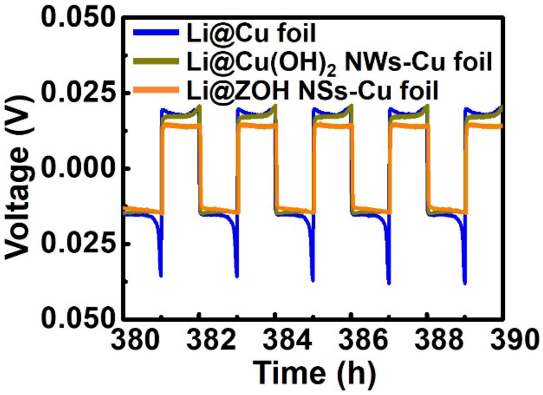


**Figure S17.** Voltage–time profiles at a current density of 1 mA $cm^{-2}$ with a capacity of 1 mAh $cm^{-2}$ after Li pre-deposition of 5 mAh $cm^{-2}$.

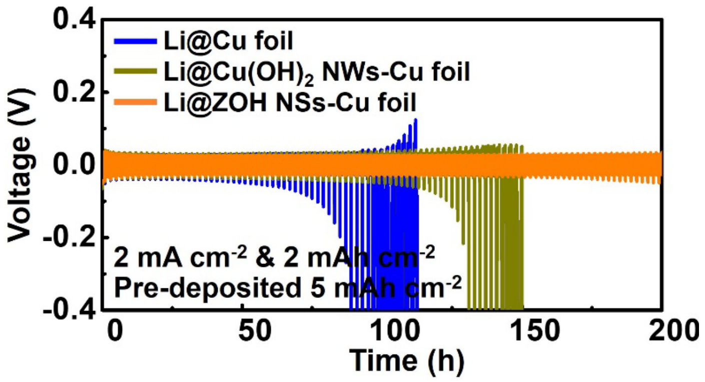


**Figure S18.** Voltage–time profiles of Li@Cu foil, Li@Cu(OH)$_2$-Cu foil, and Li@ZOH NSs-Cu foil at current density of 2 mA cm$^{-2}$ with a capacity of 2 mAh cm$^{-2}$, following Li pre-deposition of 5 mAh cm$^{-2}$.

**Table S1.** Comparison of the symmetric cell performance of Li@ZOH NSs-Cu foil with previously reported lithiophilic coatings.

| Anode | Li deposition (mAh $cm^{-2}$) | Current density (mA $cm^{-2}$) | Capacity (mAh $cm^{-2}$) | Lifespan | Ref. |
|---|---|---|---|---|---|
| **Li@ZOH NSs-Cu foil** | **5** | **1** | **1** | **1400** | **This work** |
| Zn/ZnO/$Zn(CN)_2$@C | 5 | 1 | 1 | 900 | S1 |
| 3D Zn/Cu | 10 | 1 | 1 | 950 | S2 |
| Li/MT/Cu-12h | 4 | 1 | 1 | 600 | S3 |
| $Cu_2S$ NWs/Cu | 5 | 1 | 1 | 140 | S4 |
| S-COF@Li | N/A | 1 | 1 | 600 | S5 |
| LZ-rGO | Molten Li | 1 | 1 | 1200 | S6 |
| Li-CC@MnO/Co/C | Molten Li | 1 | 1 | 870 | S7 |
| Li@CuM/Ag | N/A | 0.5 | 1 | 1100 | S8 |
| Li@$Cu_xO$-7 | Molten Li | 1 | 1 | 1200 | S9 |
| Li@3D CuO/CuS-NS | N/A | 1 | 1 | 1000 | S10 |
| Li-u$Co_3O_4$@NF | N/A | 1 | 1 | 1100 | S11 |


[S1] M. Song, Y. Li, L. Gao, R. Zhao, Y. Xu, S. Han, J. Zhu, L. Wang, Y. Zhao, *Small* **2024**, 20, 2306187.

[S2] X. Du, X. Tan, Y. Zhou, R. Xiao, X. Ke, *Ionics* **2024**, 30, 3145.

[S3] Y. Wang, Z. Chu, Y. Wang, X. Liu, Q. Zhao, *J. Alloy. Compd*. **2024**, 971, 172780.

[S4] Z. Huang, C. Zhang, W. Lv, G. Zhou, Y. Zhang, Y. Deng, H. Wu, F. Kang, Q.-H. Yang, *J. Mater. Chem. A* **2019**, 7, 727.

[S5] W. Wang, Z. Yang, Y. Zhang, A. Wang, Y. Zhang, L. Chen, Q. Li, S. Qiao, *Energy Storage Mater*. **2022**, 46, 374.

[S6] X. Zhou, S. Wang, Y. Li, Y. Yang, X. Xiao, G. Chen, *Adv. Energy Mater*. **2025**, 15, 2403640.

[S7] C. Sun, J. Lu, X. Guo, Y. Zhou, M. Wang, X. Qiu, Q. Wang, R. Yang, T. Wei, *J. Power Sources* **2024**, 607, 234597.

[S8] W. Chen, S. Li, C. Wang, H. Dou, X. Zhang, *Energy Environ. Mater*. **2023**, 6, e12412.

[S9] Y. Nie, X. Dai, J. Wang, Z. Qian, Z. Wang, H. Guo, G. Yan, D. Jiang, R. Wang, *J. Energy Chem*. **2022**, 75, 285.

[S10] C. Sun, L. Gao, W. Rong, R. Kang, Y. Yang, J. Li, Y. Bai, X. Tian, X. Bian, *Chem. Eng. J*. **2024**, 479, 147821

[S11] Y. Wang, X. Li, L. Wu, J. Tan, G. Liu, C. Ye, L. Ma, Z. Liu, M. Ye, J. Shen, *Energy Storage Mater*. **2024**, 66, 103247.

**Table S2.** Comparison of full cell performances of Li@ZOH NSs-Cu foil with other literature.

| Current collector | Modification | LFP areal capacity (mAh cm$^{-2}$) | N/P ratio | Current density (C) | Cycle number | Capacity retention | Ref. |
|---|---|---|---|---|---|---|---|
| Cu foil | **ZOH NSs-Cu foil** | **1.59** | **1.9** | **1.9** | **350** | **>90%** | **This work** |
| | $Ti_3C_2T_x$/g-$C_3N_4$ | 0.64<br>2.2 | 2.5<br>1 | 0.5<br>1 | 320<br>150 | 85.5%<br>83.8% | S12 |
| | S-CuNW | N/A | 7 | 1 | 200 | 75.4% | S13 |
| | Zn/ZnO/Zn$(CN)_2$@C | 1.36 | N/A | 1 | 200 | 94.3% | S1 |
| | Ag@Cu | 0.4 | 25 | 1 | 300 | 81% | S14 |
| | Zn/Cu | 0.61 | ≈16.4 | 1 | 100 | 90% | S2 |
| | OA-MOF | 1.7 | - | 0.5 | 200 | >80% | S15 |
| | MT/Cu | 1.4 | ≈2.9 | 1 | 200 | 98.3% | S3 |
| | BTA-Cu | 1.5 | 2 | 1 | 220 | ≈30% | S16 |
| | CF@NCZ | 1.2 | ≈39.2 | 1 | 300 | ≈83% | S17 |
| | CL-matrix@$Li_2O$ | 0.85 | ≈3.5 | 0.5 | 150 | 90% | S18 |
| | 3D porous Cu | 0.15 | ≈26.67 | 0.5 | 150 | 80% | S19 |
| | LTO@Cu | 0.33 | 3.07 | 1 | 200 | 99.5% | S20 |
| | Cu-MPA | 0.32 | 9.29 | 1 | 250 | - | S21 |
| | FGO@Cu | 0.2 | ≈14.7 | 0.5 | 100 | 88.4% | S22 |
| | CoO-LIG | 0.5 | 10 | 1 | 150 | >90% | S23 |
| Cu foam | 3D Cu@InSb | 1.45 | ≈2.8 | 1 | 50 | 90% | S24 |
| | MCNCF | 0.68 | ≈7.4 | 1 | 250 | ≈99% | S25 |
| | $ZnF_2$-Au@CF | 1.33 | 3 | 1 | 200 | 91.8% | S26 |
| | 1D@3D-Cu/Sb | 0.4 | 11.25 | 0.5 | 200 | 81.4% | S27 |
| | m-LPA foam | 0.34 | 29.4 | 0.5 | 150 | 94.4% | S28 |
| | $Cu_2S$ NWs/Cu | 0.43 | ≈4.7 | 0.5 | 100 | 96.5% | S4 |
| | $Cu_2Se$/Cu2O@3D Cu | 1.7 | ≈5.9 | 0.5 | 300 | 85.9% | S29 |
| others | Li@GDD-CH | 1.5 | 1.6 | 1 | 315 | 86.3% | S30 |
| | HP-Cu@Sn | 0.03 | ≈166.7 | 0.5 | 200 | 92.9% | S31 |
| | 3D porous current collector | 0.31 | ≈3.2 | 0.5 | 300 | 89.7% | S32 |


[S1] M. Song, Y. Li, L. Gao, R. Zhao, Y. Xu, S. Han, J. Zhu, L. Wang, Y. Zhao, *Small* **2024**, 20, 2306187.

[S2] X. Du, X. Tan, Y. Zhou, R. Xiao, X. Ke, *Ionics* **2024**, 30, 3145.

[S3] Y. Wang, Z. Chu, Y. Wang, X. Liu, Q. Zhao, *J. Alloy. Compd*. **2024**, 971, 172780.

[S4] Z. Huang, C. Zhang, W. Lv, G. Zhou, Y. Zhang, Y. Deng, H. Wu, F. Kang, Q.-H. Yang, *J. Mater. Chem. A* **2019**, 7, 727.

[S12] F. Zhao, P. Zhai, Y. Wei, Z. Yang, Q. Chen, J. Zuo, X. Gu, Y. Gong, *Adv. Sci*. **2022**, 9, 2103930.

[S13] P. Zou, C. Wang, J. Qin, R. Zhang, H. L. Xin, *Energy Storage Mater*. **2023**, 58, 176.

[S14] D. Zhang, Z. Xia, Z. Li, H. Wang, G. Zhang, Z. He, H. Zhou, M. Xu, X. Wei, W. Li, *ACS Appl. Energy Mater*. **2023**, 6, 6338.

[S15] Q. Wu, Y. Zheng, X. Guan, J. Xu, F. Cao, C. Li, *Adv. Funct. Mater*. **2021**, 31, 2101034.

[S16] T. Kang, J. Zhao, F. Guo, L. Zheng, Y. Mao, C. Wang, Y. Zhao, J. Zhu, Y. Qiu, Y. Shen, *ACS Appl. Mater. Interfaces* **2020**, 12, 8168.

[S17] J. Zhang, T. Chen, M. Chen, P. Zhang, Z. Wu, Y. Zhong, X. Guo, B. Zhong, X. Wang, *Ind. Eng. Chem. Res*. **2022**, 61,7303.

[S17] Z. Gong, C. Lian, P. Wang, K. Huang, K. Zhu, K. Ye, J. Yan, G. Wang, D. Cao, *Energy Environ. Mater*. **2021**, 5, 1270.

[S18] Y. Shi, Z. Wang, H. Gao, J. Niu, W. Ma, J. Qin, Z. Peng, Z. Zhang, *J. Mater. Chem. A* **2019**, 7, 1092.

[S20] X. Xiong, W. Yan, Y. Zhu, L. Liu, L. Fu, Y. Chen, N. Yu, Y. Wu, B. Wang, R. Xiao, *Adv. Energy Mater*. **2022**, 12, 2103112.

[S21] S. Huang, C. Meng, H. Chen, Z. Jiao, J. Qiu, H. Zhu, F. Shi, A. Yuan, H. Zhou, *Electrochim. Acta* **2024**, 482, 143998.

[S22] Y. Li, J. Xiang, Y. Li, L. Zhang, H. Tao, X. Yang, *RSC Adv*. **2024**, 14, 11089.

[S23] Z. Li, Z. Li, X. Liu, J. Wu, C. Li, C. Wang, *Electrochim. Acta* **2025**, 525, 146109.

[S24] S. Wang, C. Liu, M. Zhao, R. Song, Y. Lu, L. Gou, F. Gong, X. Fan, D Li, *J. Power Sources* **2024**, 614, 234960.

[S25] X. Wang, Z. Chen, X. Xue, J. Wang, Y. Wang, D. Bresser, X. Liu, M. Chen, S. Passerini, *Nano Energy* **2025**, 133, 110439.

[S26] K. Huang, S. Song, Z. Xue, X. Niu, X. Peng, Y. Xiang, *Energy Storage Mater*. **2023**, 55, 301.

[S27] X. Fu, C. Shang, G. Zhou, X. Wang, *J. Mater. Chem. A* **2021**, 9, 24963.

[S28] P. Xu, X. Lin, X. Hu, X. Cui, X. Fan, C. Sun, X. Xu, J.-K. Chang, J. Fan, R. Yuan, B. Mao, Q. Dong, M. Zheng, *Energy Storage Mater*. **2020**, 28, 188.

[S29] G. Guo, K. Zhang, K. Zhu, P. Yang, Z. Shao, S. Liu, L. Lin, W. Zhuang, P. Xue, Q. Zhang, Y. Yao, *Adv. Funct. Mater*. **2024**, 34, 2402490.

[S30] Y. Zhang, M. Yao, T. Wang, H. Wu, Y. Zhang, *Angew. Chem., Int. Ed*. **2024**, 63, 202403399.

[S31] Z. Luo, C. Liu, Y. Tian, Y. Zhang, Y. Jiang, J. Hu, H. Hou, G. Zou, X. Ji, *Energy Storage Mater*. **2020**, 27, 124.

[S32] Q. Yun, Y. B. He, W. Lv, Y. Zhao, B. Li, F. Kang, Q. H. Yang, *Adv. Mater*. **2016**, 28, 6932.
